# Effects of different proteases enzymatic extraction on the lipid yield and quality of Antarctic krill oil

**DOI:** 10.1002/fsn3.1017

**Published:** 2019-06-25

**Authors:** Linlin Wang, Feng Yang, Yali Rong, Yuan Yuan, Yatao Ding, Wenzheng Shi, Zhihe Wang

**Affiliations:** ^1^ College of Food Science and Technology Shanghai Ocean University Shanghai China; ^2^ National R&D Branch Center for Freshwater Aquatic Products Processing Technology (Shanghai) Shanghai China

**Keywords:** enzymatic extraction, lipid yield, protease, quality

## Abstract

This study was investigated the effects of six proteases (papain, compound proteinase, acidic protease, neutrase, pancreatin, and alcalase) on the lipid yield and quality of krill oil. The result shown that the krill oil extracted by alcalase and compound proteinase led to comparatively higher lipid yields (5.29% and 4.90%, respectively), Content of tocopherols and vitamin A, the content of omega‐3 polyunsaturated fatty acids (PUFAs) and phospholipids extracted by alcalase was relatively higher. Control and alcalase had comparatively higher concentration of astaxanthin. On the whole, compared with the extraction of solvent, enzymatic hydrolysis could improve the quality and the lipid yield of krill oil. Therefore, enzymatic hydrolysis could be used as a better method to extract krill oil.

## 1 INTRODUCTION

Antarctic krill (*Euphausia superba*) is a keystone species of Antarctic ecosystems, acting as major prey item for most of the megafauna such as baleen whales, squid, crabeater seals and brush‐tailed penguins, and other vertebrates (Cleary, Durbin, & Casas, 2018). Recently, Antarctic krill has attracted increasing attention due to its huge biomass and potential fishery resources and its special status in the Antarctic marine ecosystem (Brierley, Fernandes, & Brandon, 2002; Nicol, 2000; Pakhomov, Atkinson, & Meyer, 2004). The total number of krill was estimated to be 379 million tons (Atkinson, Siegel, Pakhomov, Jessopp, & Loeb, 2009). After harvest, krill quickly degraded, resulting in unpleasant taste and color changes due to high enzyme activity. Moreover, krill contains high fluoride content in its shell. Therefore, human consumption of whole body krill is considered to be present safety issues. And krill is mostly used for feed and bait, only a small proportion used for human consumption. As a rich resource, the use of krill as human food has so far been limited. (Kim, Jung, Lee, Chun, & Kim, 2014).

Krill oil is an important product derived from Antarctic krill and is rich in omega‐3 PUFAs. The 30% ~ 65% omega‐3 PUFAs of krill oil are stored in the form of phospholipids (Phillips, Nichols, & Jackson, 2002), particularly in eicosapentaenoic acid (EPA) and docosahexaenoic acid (DHA) (Gigliotti, Davenport, Beamer, Tou, & Jaczynski, 2011). The clinical trials of the krill oil are increasing, and most of the studies have proved that the existence form of omega‐3 PUFAs molecules in the Antarctic krill oil (glycerol ester type, ethyl ester type, and phospholipid type) played an extremely important role in its biological effects and in vivo distribution (Wael, 2008). In addition, there are also astaxanthin, tocopherols, vitamin A, and other active substances in krill, which can further improve the economic value (Xie et al., 2017). Some studies have shown that Antarctic krill oil could inhibit fatty liver and had the activity of reducing blood lipids and blood sugar (Ferramosca et al., 2012; Tandy et al., 2009; Zhu, Shi, Qian, Cai, & LI, 2008).

At present, the extraction methods of Antarctic krill oil mainly include organic solvent extraction method, supercritical CO_2_ extraction method, and enzymatic hydrolysis method (Gigliotti et al., 2011; Weng, Tao, Wang & Jin, 2012). The enzymatic hydrolysis method is an emerging oil extraction process in recent years (Zhou et al., 2013). Papain, compound proteinase, acidic protease, neutrase, pancreatin, and alcalase are the common protease in the market; these proteases prices are not too high, and some of these proteases are already used to extract oils from aquatic products. Enzymatic hydrolysis mainly uses enzymes to act on specific structures to degrade oil cells and destroy the combination of proteins and fats, and to release oil. This method does not affect other structures except enzymes. It can effectively maintain the original structure, the nutritional value, and functions of proteins and extend the shelf life of oil (Ma et al., 2010; Paul & Hauck, 2006).

In short, Antarctic krill are abundant and low in price, but because they are prone to degradation after harvest and contain high contents of fluorine, most of them are used for feed, which are rarely consumed by humans. In addition, krill oil is rich in nutrition and enzymatic hydrolysis has many advantages. After extracting, the residue can also be made into feed. Despite much research been done on the extraction method of Antarctic krill oil, effects of different proteases enzymatic extraction on phospholipids, astaxanthin and fatty acids, vitamin A, tocopherols, iodine value, and acid value have not been studied. This study will help to fill that gap. The purpose of the present study was to explain the effects of six used proteases (papain，compound proteinase, acidic protease, neutrase, pancreatin, and alcalase) on the lipid yield and quality of the oil extracted from Antarctic krill, sample of not added protease as a control. The results will be beneficial to deeply understand the effect of different extraction proteases on the quality of krill oil and can be helpful to exploit tailored krill oils having targeted functionalities and provide a strong basis for the selection of protease to extraction krill oil and provide reference for improving the extraction rate and added value of Antarctic krill oil.

## 2 MATERIALS AND METHODS

### 2.1 Materials

The samples of Antarctic krill were provided by Dalian Marine Fisheries Group Cop.of Liaoning province (Dalian, Liaoning, China), which were transported to the laboratory at −20°C and stored at −50°C until use.

The standard mixtures of 37 fatty acid methyl esters (FAMEs), retinol and α‐, β‐, γ‐, and δ‐tocopherols (purity > 95%) were purchased from Shanghai ANPEL Science Instrument Co., Ltd. (Shanghai, China). Compound proteinase and acidic protease were purchased from Shanghai Yuan Ye Biotechnology Co., Ltd. (Shanghai, China). Alcalase and neutrase were purchased from JiangSu RuiYang BioTech (Jiangsu, China). Papain, pancreatin and anhydrous ethanol, petroleum ether (30~60°C) were purchased from Sinopharm Chemical Reagent Co., Ltd. (Shanghai, China).

### 2.2 Lipid extraction

Papain, compound proteinase, acidic protease, neutrase, pancreatin, and alcalase were used as extraction proteases. 50 g of krill was homogenized with 50 ml distilled water, adjusted to the corresponding condition (papain 50°C pH 7.0, compound proteinase 50°C pH 7.0, acid protease 50°C pH 3.0, neutrase 50°C pH 7.0, pancreatin 50°C pH 7.0, and alcalase 55°C pH 8.0) in a water bath, added 0.4 g protease, and hydrolyzated 3 hr with stirring every 30min. After enzymatic hydrolysis extracted krill oil using 30 ml anhydrous ethanol, 30 minutes later extracted using 60 mL petroleum ether (30 ~ 60°C) for 30min, repeated the solvent extraction once and made a control that was not added protease at room temperature. The upper organic layer was centrifuged at 9.794 m/s^2^ for 10 min at 4°C, and then, the solvent was removed in a rotary evaporator at 60°C.

### 2.3 Analysis of lipid yield

Weighed the initial Antarctic krill (wet base) and the extracted krill oil. The lipid yield is defined as follows:

$$\text{Lipid yield}\;\left(\%\right)\frac{m}{y}=\times 100$$

where m is the weight of krill oil (g) and y is the mass of initial Antarctic krill (g).

### 2.4 Analysis of phospholipid content in krill oil

The phospholipid content was determined by the method of Avalli and Contarini (2005) with slight modifications by a high‐performance liquid chromatography (HPLC) (W2690/5, Waters, USA) equipped with a diode array detector (DAD) (Waters, USA). In brief, 0.1 g krill oil was dissolved in 1 ml chloroform:methanol (2:1, v:v), ten microliters of the product was injected into HPLC for analysis.

### 2.5 Analysis of astaxanthin content in krill oil

Using a high‐performance liquid chromatography (HPLC) (W2690/5, Waters, USA) equipped with a diode array detector (DAD) (Waters, USA) and a C18 column (5μm, 4.6 × 250 mm; Shanghai ANPEL Science Instrument Co., Ltd, Shanghai, China) with a mobile phase, containing dichloromethane:acetonitrile:methanol (20:70:10, v/v/v) under isocratic condition with a flow rate of 1.0 ml/min to analyze astaxanthin, this method was determined by Sun et al. (2018) with slight modifications. Briefly, 0.5 g of krill oil mixed with 5 ml dichloromethane and then added 0.1 mol/L sodium hydroxide (NaOH) in methanol at 4°C for 9 hr in darkness for complete saponification. The determined wavelengths were 476 nm for astaxanthin and were identified and quantified by comparing with the standards (Shanghai Macklin Biochemical Co., Ltd, Shanghai, China).

### 2.6 Fatty acids composition analysis

FAME was prepared and analyzed according to the previously developed procedure (Xie et al., 2018) with small modifications to the process of transesterification and oven temperature program. Briefly, 0.1 g krill oil mixed with 5 ml of 0.1 mol/L sodium hydroxide (NaOH) in methanol was transesterified with stirring in a 80°C water bath for 15 min and added 3 ml 14% boron trifluoride–methanol solution for further transesterification at 80°C for 5 min. After cooled to room temperature, 2 ml n‐hexane and 5 ml saturated sodium chloride solution were added, respectively. The upper organic layer was filtered through a 0.22‐um membrane and then stored at −20°C for further analysis.

The analysis of FAME samples was performed by a gas chromatography (GC) (TRACE GC Ultra, Thermo Fisher Scientific and Technology Co., Ltd., China) equipped with a hydrogen flame ionization detector (FID), automatic sample injector along with a separation column namely Trace Agilent SP‐2560 capillary column (100 m × 0.25 mm × 0.2 µm; ANPEL, shanghai, China). The other conditions were as follows: oven temperature program, 70 (3min) to 140°C (50°C/min, 2 min), 140 (10min) to 180°C (4°C/min, 2min), 180(15min) to 225°C (3°C/min, 30 min), detector temperature and inlet temperature (250°C), and split ratio (45:1). The FAME samples were identified by comparing the retention times and relative area percentages of sample peaks with those of the corresponding FAME mixture standards. The fatty acid contents were expressed as the weight (g/100g) of the total fatty acids.

### 2.7 Analysis of tocopherols and vitamin A contents and composition in krill oil

Using a high‐performance liquid chromatography (HPLC) (Agilent 1260, Shanghai Zhiyan Scientific Instrument Co., Ltd.，China) equipped with an ultraviolet detector (UV detector) (Agilent 1260，Shanghai Zhiyan Scientific Instrument Co., Ltd.，China) to analyze tocopherols and vitamin A, this method was determined by Xie et al. (2017) with slight modifications: In brief, saponification reaction of the krill oil l sample (0.5 g) was achieved using 15 mg of ascorbic acid and 5 ml of 2 mol/L KOH in ethanol at 60°C for 60min. After cooling to room temperature, 5 ml of 1.5 mg/100 ml butylated hydroxytoluene (BHT) in n‐hexane was added to extract the unsaponifiables three times, combined the mixture, and evaporated the n‐hexane to achieve extracts. The extracts were dissolved in 10 ml of n‐hexane. 20 μl of the product was injected into HPLC, and the C18 column was purchased from Shanghai ANPEL Science Instrument Co., Ltd. (Shanghai, China). The determined wavelengths of tocopherols and vitamin A were 295 nm and 325 nm, respectively. The two compounds were identified and quantified by comparison with standard products.

### 2.8 Analysis of Iodine value and Acid value in krill oil

Iodine value is the measure of the degree of unsaturation of fat and oil, which indicates the amount of absorbed iodine. The determination of iodine value was tested according to the procedure described by Barlow, Bimbo, Miller, Thorisson, and Walters (1997).

Acid value is also called acid number, is expressed as the number of milligrams of potassium hydroxide (KOH) neutralized by the free acid present in 1 g of the substance, and determination of acid value is the same as method described by Cong et al. (2007).

## 3 RESULTS AND DISCUSSION

### 3.1 Lipid yield %

**Figure 1:**
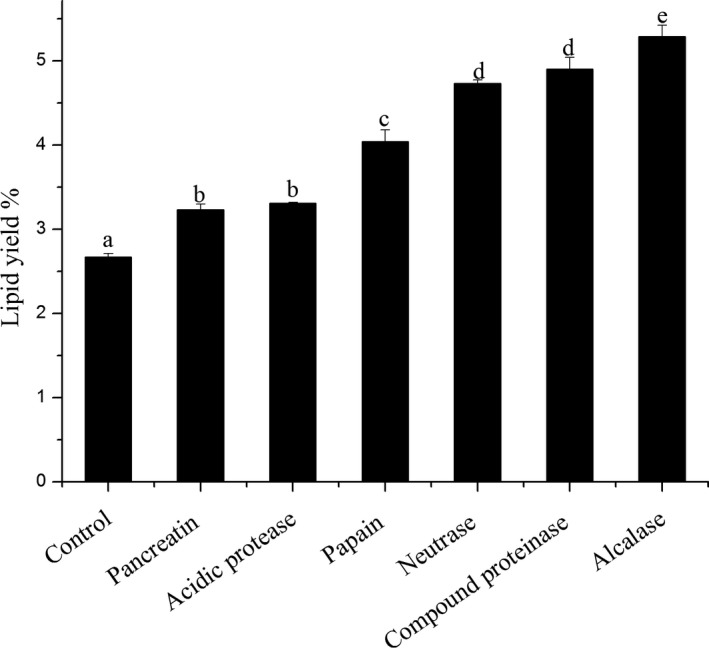
Lipid yield of different proteases for extraction from Antarctic krill

The lipid yields obtained by various proteases are presented in Figure 1. It could be observed that the lipid yields varied from 3.23%to 5.29% and they were all higher than not added protease of 2.67%. This shows that the lipid yields extracted by enzymatic hydrolysis are 1.21 to 1.98 times of by solvent and are higher than research of xu (2016) of the yield of Antarctic krill oil reached 3.89%. The yields were pancreatin＜acidic protease＜papain＜neutrase＜compound proteinase＜alcalase. The enzymolysis of protease to protein is mainly associated with the specificity of the enzyme substrate. Neutrase, compound proteinase, and alcalase can hydrolyze the peptide bond of the Antarctic krill protein better and then destroy the combination of protein and oil and release the oil. Therefore, the yield of the krill oil is high. Also, observation can be drawn that the lipid yield of neutrase and compound protease was 4.73% and 4.90%, respectively, and there was no significant difference (*p* < 0.05). This indicates that neutrase, compound proteinase, and alcalase can be used as a good protease for extracting Antarctic krill oil.

### 3.2 Phospholipid analysis

**Figure 2:**
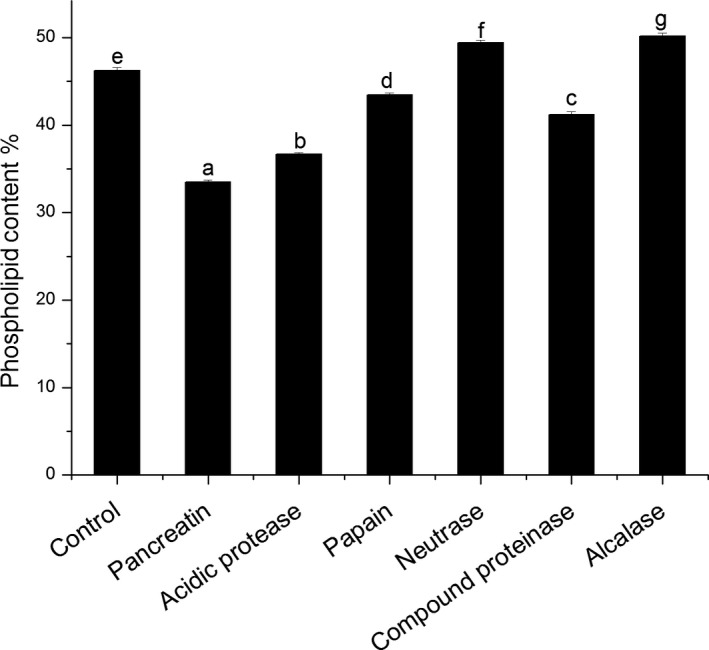
Comparison of phospholipids in Antarctic krill oil extracted with different proteases

The total phospholipid content is shown in Figure 2 presented as wt % of krill oil. Phospholipid is an important lipid class in krill oil. In the study, the content of phospholipid in krill oil in the range of 33.49%–50.19% and extracted by different proteases was different, content of phospholipid extracted by alcalase was the highest, which was 50.19%, followed by neutrase, which was 49.45%. Compared with, influenced by, the type of extraction solvent varied from 16.5% to 34.5% (Sun et al., 2018), the content of phospholipids in krill oil extracted by enzymatic hydrolysis is higher.

### 3.3 Astaxanthin analysis

Astaxanthin in Antarctic krill oil is currently recognized as the most active and safe bioactive substance. It is the most important carotenoid pigment in Antarctic krill oil, and it is also the natural strongest antioxidant and free radical scavenging in nature Pigment, which has many physiological functions such as antioxidation and tumor prevention. (Anderson, 2005; Kurashige, Okimasu, Inoue, & Utsumi, 1990; Maleyeff & Kaminsky, 2003; Maoka, Katsuyama, Kaneko, & Matsuno, 1985). Study has shown that the content of astaxanthin was in the range of 6.94–21.59 ppm (Kim et al., 2014) and astaxanthin content in krill tissue was reported in 1.5–2.0 mg/100g (Tou, Jaczynski, & Chen, 2010).

**Figure 3:**
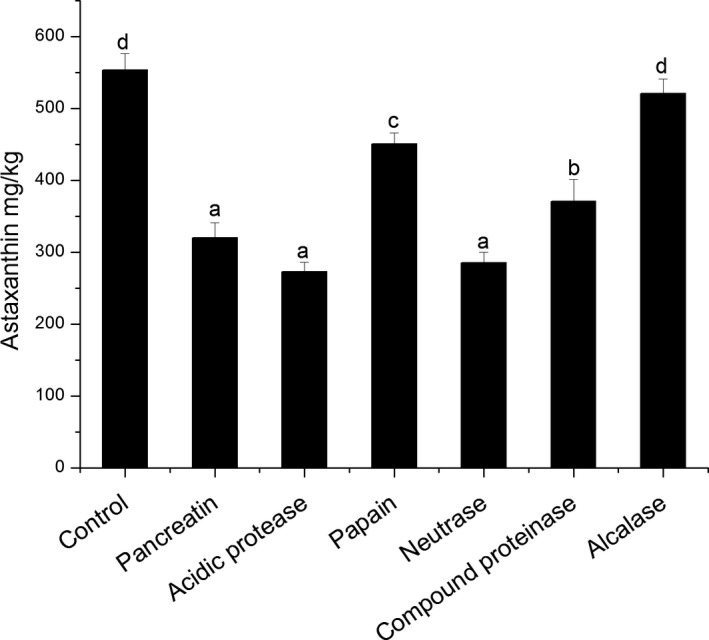
Comparison of astaxanthin in Antarctic krill oil extracted with different proteases

The astaxanthin content of the krill oil extracted by various proteases varied from 272.75 to 553.45mg/kg, as shown in Figure 3. The content of astaxanthin in the Antarctic krill oil extracted by different proteases was different. The highest concentration of astaxanthin extracted by control and alcalase was 553.45 and 520.75 mg/kg, respectively, followed by the papain, which was 450.50 mg/kg, and there was no significant difference (*p* < 0.05). Compared with the content of 93.87–222.64 mg/kg extracted by solvents (Xie et al., 2018), the astaxanthin content was higher by enzymatic hydrolysis.

### 3.4 Fatty acids composition analysis

Contents of fatty acids in krill oil are listed in Table 1, C14:0, C15:1, C16:1, C18:1, C20:5 (EPA), and C22:6 (DHA). EPA and DHA are the main fatty acids in Antarctic krill oil. According to the other studies and findings described (Kolakowska, Kolakowski, & Szczygielski, 1994), the n‐3 PUFA, EPA, and DHA are especially abundant in krill oil, which was due to Antarctic krill consuming single‐cell marine microalgae.

**Table 1:** Fatty acid composition of Antarctic krill oil extracted with different proteases†

Fatty acids	Control	Pancreatin	Acidic protease	Papain	Neutrase	Compound proteinase	Alcalase
C14:0	9.05 ± 0.49^a^	8.88 ± 0.14^a^	8.18 ± 0.02^a^	12.37 ± 2.90^b^	10.49 ± 1.52^ab^	9.10 ± 0.01^a^	12.37 ± 0.12^b^
C14:1	0.05 ± 0.01^b^	0.02 ± 0.00^a^	0.02 ± 0.00^a^	0.04 ± 0.01^b^	0.07 ± 0.00^c^	0.08 ± 0.02^c^	0.08 ± 0.00^c^
C15:0	0.20 ± 0.01^c^	0.05 ± 0.00^a^	0.07 ± 0.01^a^	0.26 ± 0.02^d^	0.10 ± 0.00^b^	0.19 ± 0.00^c^	0.11 ± 0.00^b^
C15:1	8.42 ± 0.25^ab^	7.72 ± 0.23^a^	12.67 ± 0.33^d^	10.76 ± 0.01^c^	10.59 ± 0.46^c^	8.89 ± 0.56^b^	12.58 ± 1.58^d^
C16:1	11.83 ± 0.68^cd^	6.02 ± 0.27^a^	8.90 ± 0.07^b^	12.87 ± 0.40^d^	6.95 ± 0.82^a^	10.73 ± 0.69^c^	12.22 ± 0.27^d^
C17:1	0.02 ± 0.00^ab^	0.02 ± 0.00^b^	0.03 ± 0.00^c^	0.02 ± 0.00^a^	0.02 ± 0.00^b^	0.02 ± 0.00^b^	0.04 ± 0.00^d^
C18:0	1.50 ± 0.18^a^	1.90 ± 0.29^ab^	3.38 ± 0.46^c^	1.93 ± 0.07^ab^	2.74 ± 0.75^b^	1.75 ± 0.15^a^	3.04 ± 0.76^c^
C18:1N9T	5.79 ± 0.00^a^	6.20 ± 0.23^a^	8.39 ± 0.27^b^	10.83 ± 0.05^d^	8.54 ± 0.44^b^	10.03 ± 0.31^c^	13.55 ± 0.22^e^
C18:1N9C	11.43 ± 0.33^b^	9.07 ± 0.23^a^	12.45 ± 0.38^bc^	13.88 ± 0.02^d^	12.60 ± 0.28^c^	12.30 ± 0.96^bc^	14.50 ± 1.32^d^
C18:2N6C	0.47 ± 0.03^c^	0.67 ± 0.02^d^	0.24 ± 0.04^a^	0.36 ± 0.03^b^	1.04 ± 0.04^f^	0.28 ± 0.01^a^	0.86 ± 0.11^e^
C18:3(*n*−3)	0.48 ± 0.00^b^	0.71 ± 0.00^c^	0.28 ± 0.00^a^	0.50 ± 0.00^b^	0.55 ± 0.03^b^	0.49 ± 0.04^b^	1.02 ± 0.09^d^
C20:1	1.00 ± 0.15^e^	0.11 ± 0.00^ab^	0.07 ± 0.01^a^	0.83 ± 0.03^d^	0.23 ± 0.02^bc^	0.76 ± 0.08^d^	0.35 ± 0.00^c^
C20:2	1.65 ± 0.18^c^	1.29 ± 0.00^b^	0.39 ± 0.03^a^	1.95 ± 0.04^d^	1.21 ± 0.15^b^	1.92 ± 0.08^d^	2.27 ± 0.00^e^
C22:1N9	0.54 ± 0.02^c^	0.45 ± 0.01^b^	0.15 ± 0.01^a^	0.66 ± 0.04^d^	0.55 ± 0.05^c^	0.54 ± 0.03^c^	1.04 ± 0.00^e^
C22:2	0.09 ± 0.00^e^	0.08 ± 0.01^c^	0.06 ± 0.01^b^	0.02 ± 0.00^a^	0.09 ± 0.01^d^	0.08 ± 0.00^c^	0.09 ± 0.00^de^
C20:5(*n*−3)	21.22 ± 0.37^e^	18.70 ± 0.35^a^	20.05 ± 0.00^c^	18.75 ± 0.00^a^	20.78 ± 0.59^d^	19.80 ± 0.09^b^	21.22 ± 1.59^e^
C22:6(*n*−3)	10.69 ± 0.45^e^	8.55 ± 0.04^d^	5.27 ± 0.00^a^	8.05 ± 0.00^c^	8.65 ± 0.06^d^	6.20 ± 0.11^b^	10.69 ± 0.69^e^
∑SFA	10.14 ± 0.62^e^	10.23 ± 0.41^c^	10.99 ± 0.44^a^	13.73 ± 2.69^d^	12.60 ± 1.75^e^	10.42 ± 0.15^b^	14.65 ± 0.61^e^
∑MUFA	37.10 ± 1.32^b^	28.12 ± 0.24^a^	40.51 ± 0.52^c^	47.39 ± 0.34^d^	37.56 ± 1.15^b^	41,90 ± 2.38^c^	51.62 ± 0.20^e^
∑PUFA	33.01 ± 0.94^e^	28.70 ± 0.40^c^	25.03 ± 0.02^a^	28.33 ± 0.00^c^	30.82 ± 0.72^d^	27.51 ± 0.24^b^	34.49 ± 0.02^f^
∑PUFA(*n*−3)	30.90 ± 0.80^e^	26.75 ± 0.37^c^	24.38 ± 0.00^b^	26.09 ± 0.00^b^	26.78 ± 0.64^d^	25.33 ± 0.15^a^	31.42 ± 0.08^f^

Note

MUFA, monounsaturated fatty acids; PUFA, polyunsaturated fatty acids; SFA, saturated fatty acids.

Different superscript letters in a row indicate significant differences for individual fatty acid (*p* < 0.05), the same below.

† Values are means ± standard deviation and are expressed as mass g/100g

Table 1 shows the content of fatty acids extracted by different proteases was different, and this phenomenon may be related to its extraction principle. Compared with the research of Sidhu (2010) of 23.4%, the content of PUFA (25.03–34.49 g/100g) in krill oil extracted by enzymatic hydrolysis is relatively higher, and compared with the extraction of organic solvents (24.24%–33.05%) (Xie et al., 2017), the content of n‐3 PUFA (24.38–31.42 g/100g) in krill oil extracted by enzymatic hydrolysis is relatively higher and the content of PUFA and MUFA is also higher. In addition to control and compound proteinase, the contents of fatty acids and n‐3 PUFA were positively associated with lipid yield of krill oil, so we can conclude that the content of n‐3 PUFA in the krill oil is related to the yield and enzymatic hydrolysis can better maintain the nutrition of krill oil. At the same time, it is also shown that besides the protease type, the extraction temperature and pH also have certain influence on the fatty acid composition of Antarctic krill oil, the raw materials we use are not dried, which better protects the unsaturated fatty acids from oxidation.

### 3.5 Tocopherols and vitamin A analysis

According to the reports by Suzuki and Shibata (1990), krill was rich in tocopherol and vitamin A. Two kinds of tocopherol and vitamin A in krill oil were provided detail information in this study. Table 2 shows that krill oil contained tocopherol and vitamin A of 15.50–39.28 mg/100g and 82.36–95.32 µg/100g, respectively, the content of tocopherol is mainly dominated by α‐tocopherol (14.70–38.17 mg/100 g) and γ‐tocopherol (0.54–1.11 mg/100 g), and δ‐tocopherol has not been detected.

**Table 2:** Tocopherols and vitamin A contents of Antarctic krill oil extracted with different proteases

Vitamin	Control	Pancreatin	Acidic protease	Papain	Neutrase	Compound proteinase	Alcalase
Vitamin A (µg/100g)	82.36 ± 0.17^a^	92.15 ± 0.64^e^	87.80 ± 0.20^c^	88.95 ± 0.04^d^	86.67 ± 0.36^b^	88.35 ± 0.02^cd^	95.32 ± 1.12^f^
α‐tocopherol	22.00 ± 0.04^b^	21.40 ± 0.66^b^	22.40 ± 1.46^b^	29.77 ± 0.04^c^	14.70 ± 0.16^a^	29.76 ± 0.02^c^	38.17 ± 0.85^d^
γ‐tocopherol	0.54 ± 0.01^a^	0.65 ± 0.03^b^	0.82 ± 0.01^c^	0.95 ± 0.01^d^	0.80 ± 0.03^c^	0.83 ± 0.02^c^	1.11 ± 0.04^e^
Total tocopherols (mg/100g)	22.54 ± 0.28^b^	22.04 ± 0.69^b^	23.22 ± 1.46^b^	30.71 ± 0.42^c^	15.50 ± 0.15^a^	30.57 ± 0.28^c^	39.28 ± 0.89^d^

Values are means ± standard deviation. Different superscript letters in a row indicate significant differences for individual fatty acid (*p* < 0.05).

In the present study, the contents of vitamin A and tocopherol obtained by enzymatic hydrolysis of alcalase were higher, which was 95.32 µg/100g and 39.28 mg/100g, respectively, lower than 29.39 mg/100 g of tocopherols and higher than 34.32 mg/100 g of vitamin A of the research of Xie et al. (2018), and the contents of vitamin A and tocopherols were positively associated with lipid yield; these results provide date support for the selection of protease to hydrolyze Antarctic krill. It is proved that alcalase can be used as the best protease for extracting Antarctic krill oil compared with other proteases.

### 3.6 Iodine value and Acid value analysis

Iodine value (IV) is the measure of the degree of unsaturation of fat and oil, which indicates the amount of absorbed iodine. It is an important parameter in oil industry, which can be used as guidance for oil processing. The more the fatty acid is unsaturated, the greater the iodine value is. (Haryati, Man, Ghazali, Asbi, & Buana, 1998). As shown in Table 3, the content of unsaturated fatty acids in krill oil hydrolyzed by alcalase was the highest, followed by control, acidic protease, and papain. The effects of different proteases on the IV displayed a similar trend to that on fatty acids.

**Table 3:** Iodine value and acid value of Antarctic krill oil extracted with different proteases

	Control	Pancreatin	Acidic protease	Papain	Neutrase	Compound proteinase	Alcalase
Iodine value (g/100g)	252.95 ± 0.36^e^	69.73 ± 0.27^a^	245.56 ± 0.89^d^	244.22 ± 1.34^d^	112.69 ± 0.36^b^	127.73 ± 0.63^c^	265.92 ± 0.81^f^
Acid value (mg/g)	21.74 ± 0.99^a^	84.85 ± 0.99^e^	31.56 ± 0.98^cd^	25.95 ± 0.99^b^	25.95 ± 0.98^b^	27.35 ± 0.99^b^	29.45 ± 0.78^bc^

Values are means ± standard deviation. Different superscript letters in a row indicate significant differences for individual fatty acid (*p* < 0.05).

Acid value is also being called acid number, is expressed as the number of milligrams of potassium hydroxide (KOH) neutralized by the free acid present in 1g of the substance. As shown in Table 3, except pancreation, the freshness of the Antarctic krill oil extracted by other proteases is close to that of the control. This indicates that the enzymatic hydrolysis method has little effect on the freshness of krill oil and could increase the extraction rate; therefore, the enzymatic hydrolysis method could be used as a good method for extracting krill oil.

Compared with the research of Cao et al. (2013), iodine value of krill oil extracted by enzymatic hydrolysis is higher than extracted by mixed solvent, and acid value is lower than extracted by mixed solvent. This shows that the quality of krill oil extracted by enzymatic hydrolysis is better.

## 4 CONCLUSIONS

This study was investigated the effects of six proteases (papain, compound proteinase, acidic protease, neutrase, pancreatin, and alcalase) on the lipid yield and quality of krill oil. From this study, we can conclude that different proteases have different effects on krill oil. The results indicated that compound proteinase and alcalase were achieved higher level of lipid yields, phospholipids, tocopherols, and vitamin A. The content of omega‐3 polyunsaturated fatty acids (PUFAs) and phospholipids extracted by alcalase was relatively higher. Control and alcalase had comparatively higher concentration of astaxanthin. It was also found that the content of unsaturated fatty acids extracted from krill oil by enzymatic hydrolysis is higher. The content of PUFA and MUFA and the content of vitamin A and tocopherols were positively associated with the lipid yield of krill oil. The effects of different proteases on the IV were positively associated with the fatty acids. The results are beneficial to help deep understanding of the effect of different extraction proteases on the quality of krill oil and could be helpful to exploit tailored krill oils having targeted functionalities. The data produced in this study may help manufacturers to more appropriately select proteases in the krill oil industry.

## CONFLICT OF INTEREST

The authors declare that they do not have any conflict of interest.

## ETHICAL STATEMENT

This study does not involve any human or animal testing.
